# Simultaneous binding to the tracking strand, displaced strand and the duplex of a DNA fork enhances unwinding by Dda helicase

**DOI:** 10.1093/nar/gku845

**Published:** 2014-09-23

**Authors:** Suja Aarattuthodiyil, Alicia K. Byrd, Kevin D. Raney

**Affiliations:** Department of Biochemistry and Molecular Biology, University of Arkansas for Medical Sciences, Little Rock, AR 72205, USA

## Abstract

Interactions between helicases and the tracking strand of a DNA substrate are well-characterized; however, the role of the displaced strand is a less understood characteristic of DNA unwinding. Dda helicase exhibited greater processivity when unwinding a DNA fork compared to a ss/ds DNA junction substrate. The lag phase in the unwinding progress curve was reduced for the forked DNA compared to the ss/ds junction. Fewer kinetic steps were required to unwind the fork compared to the ss/ds junction, suggesting that binding to the fork leads to disruption of the duplex. DNA footprinting confirmed that interaction of Dda with a fork leads to two base pairs being disrupted whereas no disruption of base pairing was observed with the ss/ds junction. Neutralization of the phosphodiester backbone resulted in a DNA-footprinting pattern similar to that observed with the ss/ds junction, consistent with disruption of the interaction between Dda and the displaced strand. Several basic residues in the 1A domain which were previously proposed to bind to the incoming duplex DNA were replaced with alanines, resulting in apparent loss of interaction with the duplex. Taken together, these results suggest that Dda interaction with the tracking strand, displaced strand and duplex coordinates DNA unwinding.

## INTRODUCTION

Helicases are ubiquitous molecular motor proteins which participate in various aspects of nucleic acid metabolism such as DNA replication, recombination, repair, transcription, translation and splicing of transcripts by providing ssDNA intermediates (1–6). Helicases transduce energy from ATP hydrolysis to unwind and translocate on nucleic acids. Mutations in helicase genes involved in DNA repair processes have been linked to numerous human diseases (7–12) characterized by genomic instability, premature aging and predisposition to cancer (10,13–15). The various human diseases caused by mutant helicases suggest that multiple processes involving DNA manipulation may be defective (16). It is essential to understand the mechanisms by which helicases perform different biochemical functions so that the relationship between mutations and specific disease states can be understood at the molecular level. The possibility that the efficacy of chemotherapeutic agents could be increased by administering drugs that target helicases along with the anti-cancer drugs (17) also raises the need to study the mechanisms of helicases.

Understanding protein–DNA interactions with each strand are crucial to develop a mechanistic framework for helicases. The strand that the helicase moves along is referred to as the tracking strand and the complementary strand is the displaced strand. One of the least understood aspects of helicase-catalyzed DNA unwinding is the role of protein interactions with the displaced strand. The significance of interaction between the displaced strand and Dda helicase was suggested based on the appearance of an intermediate that occurs during unwinding (18). Since then, other studies have found similar results for different helicases (19,20).

One of the outstanding questions in helicase enzymology is whether the displaced strand follows a specific path around the enzyme (18,21). The importance of this question relates directly to several physiologically relevant activities catalyzed by helicases for which no clear mechanism has been provided. For example, helicases can catalyze annealing of two strands (22–24). Helicases can switch strands during DNA unwinding (25–27) which would appear to require interaction with the displaced strand (28). Some helicases exhibit preferences for different DNA substrates such as quadruplex or forked DNA (29–32). It is possible that these ‘non-standard’ helicase activities rely on interactions between a helicase and the displaced strand. Here, we investigated the role of the displaced strand using a combination of DNA footprinting and kinetic studies.

Extensive biochemical and structural characterization of the SF1A helicases (PcrA, Rep and UvrD) have provided an in-depth understanding of their mechanisms of action (33–38). Comparatively less is known about the SF1B helicases. Several enzymes of this family have important functions in both eukaryotes and prokaryotes. For example, Pif1 helicase plays important roles in multiple processes including regulation of telomeric DNA, Okazaki fragment processing and mitochondrial DNA maintenance (39–43). Dda, from bacteriophage T4, is the best characterized SF1B helicase and serves as a model for understanding the mechanisms of this class of helicases.

Dda helicase translocates on ssDNA (27,44–45) and unwinds duplex DNA with 5′ to 3′ directionality (46). Dda can function as a monomeric molecular motor (47). However, Dda monomers can function cooperatively to displace streptavidin bound to biotin-labeled oligonucleotides (48). The cooperativity results from multiple helicase molecules translocating in the same direction. Dda displays limited processivity while unwinding DNA (continuously dissociates and reassociates with the DNA molecule being unwound) rather than a processive translocation mechanism (46). The specific biological role for Dda activity appears to occur early during T4 phage infection and might be related to DNA replication initiation (49,50).

The X-ray crystal structure of Dda has been solved with ssDNA recently (28). The three major domains (1A, 2A and 2B) form a cleft that binds ssDNA. A smaller domain (1B) binds near the 3′-end of ssDNA and serves as a wedge or pin to separate the incoming duplex. A phenylalanine residue within domain 1B interacts by stacking with an incoming base to actively melt the duplex. Helicase motifs are located in domains 1A and 2A and have been assigned specific roles in coupling ATP hydrolysis to movement along DNA (5).

**Figure 1.**
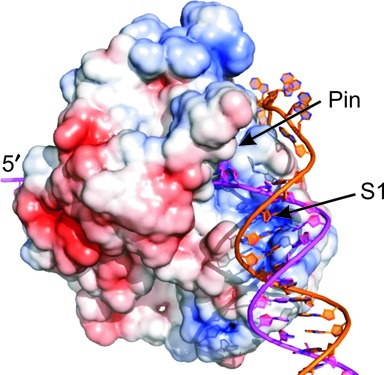
The surface view of Dda bound to duplex DNA is shown (Adapted from (28) (PDB code 3UPU)). The blue, red and white patches represent the positive, negative and neutral/hydrophobic amino acid residues, respectively. S1 is the preferred binding site for the incoming duplex DNA. The pin region separating the strands of duplex DNA is shown. Dda interacts with the translocase strand (magenta) in the known binding site and the displaced strand (orange) and duplex (magenta and orange) are proposed to interact with additional sites on the surface of the enzyme.

X-ray crystallographic studies have identified many aspects of the interactions with the translocase strand (28,35,51–52), but thus far, a specific binding site for the displaced strand has not been revealed. Crystallographic approaches may not reveal transient interactions that are required for helicase activity. In the case of Dda helicase, kinetic studies have suggested that the displaced strand interacts with the helicase, giving rise to a pause in the kinetic mechanism for unwinding (18). From previous studies, DNA unwinding (18,53) and translocation rates of Dda (27) were found to be similar (250–300 s^−1^) indicating that Dda is a highly active helicase (54). We hypothesized that Dda binds to both the translocating and displaced strands in a defined manner to enable its DNA unwinding activity (Figure 1). To test this hypothesis, we have taken two approaches. First, we studied the helicase activity of Dda in the presence of DNA substrates with or without a fork structure. Furthermore, we have employed DNA-footprinting assays using potassium permanganate (KMnO_4_) to explore binding of Dda with forked and non-forked substrates. Our results demonstrate the significance of interactions between Dda and the displaced strand.

## MATERIALS AND METHODS

### Nucleic acids and other reagents

Oligonucleotides were purchased from Integrated DNA Technologies, purified using denaturing PAGE (55) and stored in 10 mM HEPES pH 7.5 and 0.1 mM EDTA. The DNA concentrations were determined by measuring the UV absorbance at 260 nm and calculated using the extinction coefficient of the DNA (56). MeP-modified oligonucleotides were from Midland. Heparin, pyruvate kinase, lactate dehydrogenase and bovine serum albumin (BSA) were purchased from Sigma. ATP, Mg(OAc)_2_, HEPES, EDTA, KOAc, βME and glycerol were from Fisher. Poly(dT) was from Amersham Pharmacia Biotech. T4 polynucleotide kinase was purchased from Promega. [γ^32^P]ATP was from Perkin Elmer Life Sciences.

### Expression and purification of Dda helicase

Wild-type Dda and Dda mutants (Dda *K24A-K25A* and *K101A-R122A-K123A*) were overexpressed and purified as described (55). Protein concentration was determined by measuring the absorbance at 280 nm and also by the Bradford method (57). The extinction coefficient of Dda (59,010 cm^−1^ M^−1^) based on the amino acid sequence was used to quantify the protein (58).

### Analysis of the ATPase (*k*_cat_) activity of Dda using coupled spectrophotometric assay

Steady-state ATPase activity of Dda was analyzed using a coupled spectrophotometric assay (44) (Supplementary Figure S2). The reaction mixture contained 25 mM HEPES (pH 7.5), 10 mM KOAc, 10 mM Mg(OAc)_2_, 5 mM ATP, 4 mM phosphoenol pyruvate, 21.6 units/ml pyruvate kinase, 33.2 units/ml lactate dehydrogenase, 0.9 mM NADH, 2 mM βME and 100 μM poly(dT). ATP hydrolysis rates were determined by measuring the conversion of NADH to NAD^+^ at 380 nm (ϵ_380_ of NADH is 1210 M^−1^ cm^−1^). The oxidation of 1 mol of NADH corresponds to the hydrolysis of 1 mol of ATP.

### Single-turnover rapid quench unwinding experiments

**Scheme 1.**
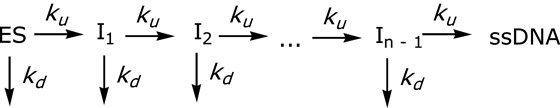
Unwinding data was fit to an n-step sequential mechanism where ES is the enzyme substrate complex, I is a partially unwound intermediate, ssDNA is the product, *k_u_* is the rate constant for unwinding, *k_d_* is the rate constant for dissociation, and n is the number of steps.

Unwinding assays were carried out using a quench-flow apparatus (RQF-3, KinTek Instruments, Austin). Substrates used for the unwinding assay are shown (Table 1). All concentrations listed are after mixing unless noted otherwise. Buffer composition was 25 mM HEPES pH 7.5, 10 mM KOAc, 0.1 mM EDTA, 0.1 mg/ml BSA and 2 mM βME. Dda and γ^32^P-labeled DNA at the concentrations indicated in the figure legends were allowed to incubate at 25°C for 3 min before adding 5 mM ATP, 10 mM Mg(OAc)_2_, 30-fold excess of an annealing trap (complementary to the displaced strand) and 100 μM poly(dT) protein trap. Poly(dT) was included in the reaction to prevent Dda from rebinding the substrate after the first catalytic turnover which ensures single-turnover conditions with respect to the DNA. The reaction was quenched at increasing times (5–500 ms) using 400 mM EDTA. Double-stranded and ssDNA were resolved by 20% (w/v) native PAGE. Radioactivity was visualized using a Typhoon Trio phosphorimager (GE Healthcare) and quantified using ImageQuant software. The fraction of ssDNA was determined as described (59). The unwinding time courses were fit using KinTek Explorer (60) to Scheme 1.

**Table 1.** DNA-unwinding substrates

DNA substrate	Sequence
7T16bp ss/ds junction	5′-TTT TTT TCG CTG ATG TCG CCT GG-3′
	3′-GC GAC TAC AGC GGA CC-5′
7T16bp fork	5′-TTT TTT TCG CTG ATG TCG CCT GG-3′
	3′-TTT TTT TGC GAC TAC AGC GGA CC-5′
14T16bp ss/ds junction	5′-T TTT TTT TTT TTT TCG CTG ATG TCG CCT GG-3′
	3′-GC GAC TAC AGC GGA CC-5′
14T16bp fork	5′-T TTT TTT TTT TTT TCG CTG ATG TCG CCT GG-3′
	3′-T TTT TTT TTT TTT TGC GAC TAC AGC GGA CC-5′
7T20bp ss/ds junction	5′-TTT TTT TCG CTG ATG TCG CCT GGT ACG-3′
	3′-GC GAC TAC AGC GGA CCA TGC-5′
7T20bp fork	5′-TTT TTT TCG CTG ATG TCG CCT GGT ACG-3′
	3′-TTT TTT TGC GAC TAC AGC GGA CCA TGC-5′

DNA substrates contained either 7 or 14 thymidines in the 5′-ssDNA overhang and 16 or 20 bps in the duplex region in order to create a ss/ds DNA junction. Another set of DNA substrates used in the study contained additional thymidines on the displaced strand creating a DNA fork. For the DNA unwinding assays, the tracking strands of the substrates were radiolabeled with γ^32^P.

The enzyme-substrate complex is converted to product in ‘*n’* identical, sequential steps, defined by the rate constant *k_u_*. At each step, the helicase can also dissociate from the DNA with a rate constant of *k_d_*. The velocity of unwinding (*V*_un_) was calculated using Equation (1).

(1) \begin{equation*} V_{{\rm un}} = k_{{\rm un}} *\frac{{L - L_0 - L_B }}{n} \end{equation*}

*L* is the number of bps in the DNA substrate duplex. *L_0_* is the number of bps that melt spontaneously due to thermal fluctuations as measured previously (18) and *L_B_* is the number of base pairs that melt due to helicase binding. ‘*n’* is the number of steps taken by the helicase to unwind the substrate. ‘*m*’ is the kinetic step size ((*L-L_0_-L_B_)/n*), i.e. the number of bps unwound between two successive rate limiting steps that are repeated in the unwinding cycle (61,62). The average unwinding rate (*V*_un_) is *k*_un_ × *m*. The observed rates of dissociation and the unwinding rates are shown in Tables 2 and 3. The unwinding data was plotted using Kaleidagraph software.

**Table 2.** Kinetic parameters determined by data analysis from Figures 2 and 6

DNA substrates	Enzyme	[DNA], nM	*k*_u_ (s^−1^)	*L-L_0_-L_B_*	(*L-L_0_-L_B_*)/*n*	*mk*_u_ (bp s^−1^)
7T16bp ss/ds junction	wtDda	2	78.4 ± 3.8	8	2.66	209
7T16bp fork	wtDda	2	71.9 ± 8.9	6	3	216
7T16bp ss/ds junction	*K24AK25A*	2	62.5 ± 2.5	8	2.66	166
7T16bp fork	*K24AK25A*	2	50.7 ± 3.7	6	3	152
14T16bp ss/ds junction	wtDda	2	63.5 ± 2.5	8	2.66	171
14T16bp fork	wtDda	2	63.4 ± 8.9	6	3	190
7T20bp ss/ds junction	wtDda	2	61.3 ± 4.3	12	4	245
7T20bp fork	wtDda	2	61.9 ± 9.3	10	5	310

The kinetic step size, (*L-L_0_-L_B_*)/*n* = m, is the number of bps unwound between two successive rate limiting steps that are repeated in the unwinding cycle. *k*_u_ is obtained by fitting the data using KinTek Explorer to Scheme 1. The number of bps unwound per second was obtained from *mk*_u_.

**Table 3.** Kinetic parameters determined by data analysis from Figure 3

DNA substrates	[DNA], nM	*k*_u_ (s^−1^)	L-L_0_-L_B_	(*L-L_0_-L_B_*)/*n*	*mk*_u_ (bp s^−1^)
7T16bp ss/ds junction	75	104 ± 10	8	2.66	277
7T16bp fork	75	93.3 ± 17.0	6	3	280
7T16bp ss/ds junction	150	105 ± 5	8	2.66	279
7T16bp fork	150	103 ± 11	6	3	309
7T16bp ss/ds junction	300	114 ± 21.7	8	2.66	305
7T16bp fork	300	141 ± 6.8	6	3	423

The kinetic step size, (*L-L_0_-L_B_*)/*n* = m, is the number of bps unwound between two successive rate limiting steps that are repeated in the unwinding cycle. *k*_u_ is obtained by fitting the data using KinTek Explorer to Scheme 1. The number of bps unwound per second was obtained from m*k*_u_.

### Potassium permanganate (KMnO_4_) footprinting

Substrates used for footprinting are shown in Table 4. Buffer composition was 25 mM HEPES pH 7.5, 0.1 mM EDTA, 10 mM KOAc, 10 mM Mg(OAc)_2_, 0.1 mg/ml BSA and 2 mM βME. γ^32^P-labeled DNA (50 nM) and Dda (500 nM) were incubated for 3 min at 25°C for the protein/DNA interaction to occur, and the footprinting reaction was initiated by addition of 5 mM KMnO_4_. The reaction was quenched using 1 M βME and 0.2 M EDTA after 5 s. Biotin-labeled γ^32^P-labeled DNA was mixed with streptavidin Dynabeads (Invitrogen) by vortexing at room temperature for 30 min. DNA was cleaved from Dynabeads by adding 1 M piperidine and 0.1 mM biotin. DNA was resolved on a 20% acrylamide, 7 M urea gel. Radioactivity was visualized using a Typhoon Trio phosphor imager (GE Healthcare) and the relative reactivity of each thymidine was determined using ImageQuant software (63).

**Table 4.**
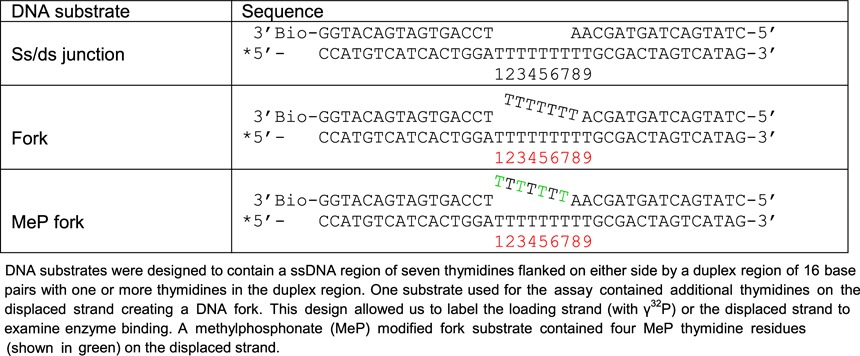
DNA-footprinting substrates

### Fluorescence titrations

Substrates used are shown in Table 5. 2-aminopurine labeled fork or ss/ds junction DNA (100 nM) was titrated with Dda in 25 mM HEPES pH 7.5, 10 mM KOAc, 0.1 mM EDTA, 0.1 mg/ml BSA, 2 mM βME in an SLM Aminco-Bowman Series 2 luminescence spectrometer maintained at 25°C with a circulating water bath. Samples were excited at 315 ± 0.5 nm and emission was measured at 380 ± 4 nm. The change in emission of samples titrated with Dda was corrected for sample dilution by subtracting the change in fluorescence of samples titrated with an equal volume of protein sample buffer. Due to the similarity in the fluorescence spectra of 2-aminopurine and tryptophan, it was also necessary to subtract the fluorescence due to Dda from the data. The fluorescence due to Dda was obtained by titrating unlabeled fork or ss/ds junction DNA (100 nM) with Dda. Data was fit to the Hill equation to obtain the Hill coefficient and the concentration of protein at which half of the maximal fluorescence was obtained (K_1/2_).

**Table 5.**
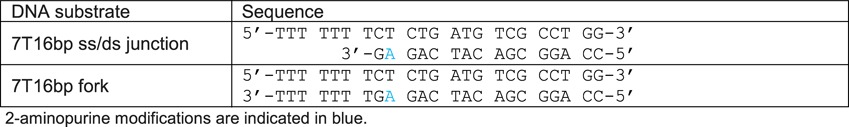
Fluorescence titration substrates

## RESULTS

### Dda helicase unwinds forked DNA with enhanced product formation compared to a ss/dsDNA junction (ss/ds junction) under excess enzyme conditions

The X-ray crystallographic structure of Dda bound to ssDNA revealed the interactions with the tracking strand (28). A model was proposed for binding to the incoming duplex DNA, which allows the tracking strand to feed directly into the DNA binding site (Figure 1). The specific path for the tracking strand was not specified, but evidence suggests that Dda can interact with the displaced strand (18). To investigate the functional significance for interaction with the duplex and the displaced strand, Dda-catalyzed duplex unwinding was carried out using substrates containing both a 5′-ssDNA overhang and a 3′-ssDNA overhang (DNA fork) or only a 5′-arm (ss/ds DNA junction).

**Figure 2.**
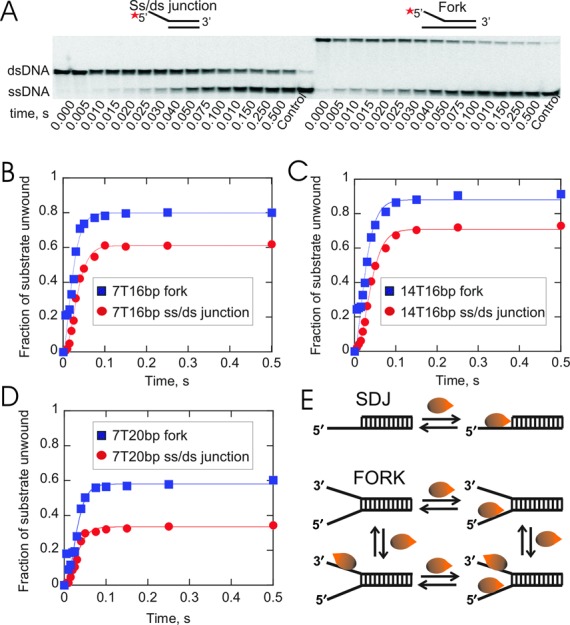
Dda efficiently unwinds forked DNA substrates when the enzyme concentration exceeds the substrate concentration. **(A)** The substrates contained either ssDNA overhangs or DNA forks. Sequences are shown in Table 1. DNA substrates (2 nM) were unwound in the presence of excess concentration of Dda (150 nM). Representative native PAGE image showing unwinding of 7T20bp ss/ds junction and forked DNA substrates. **(B–D)** Plots of ssDNA product formed over time with six different DNA substrates. The amplitude of product formation is higher for each of the forked substrates compared to the corresponding ss/ds junction substrates although unwinding rates were similar (Table 2). The ss/ds junction unwinding data were fit using KinTek Explorer to Scheme 1 for a three-step mechanism and plotted using Kaleidagraph software. The forked DNA substrates exhibited a reduced lag phase; therefore these data were ﬁt to a two-step mechanism. **(E)** Cartoon of the DNA binding mechanism under excess enzyme conditions for substrates containing 7 nt of ssDNA in the ss/ds junction or DNA fork. One molecule of Dda can bind to the 5′-overhang of the ss/ds junction substrate. Each arm of the DNA fork can bind to Dda and the relative concentration of each species is dependent on the concentrations of enzyme and substrate. Dda bound to only the 3′-arm results in a non-productive complex.

Initial DNA unwinding experiments were performed under single-turnover conditions with excess enzyme relative to DNA substrate to determine the unwinding rate and processivity. Excess enzyme conditions ensured that the ssDNA binding sites were occupied by the enzyme. Substrates were designed based on previous data indicating that Dda is unable to efficiently unwind DNA with less than 6 nucleotides (nt) of ssDNA overhang (64) and that each molecule of Dda sequesters ∼7–8 bases (28). The overhangs were 7 nt or 14 nt in length. For some unwinding experiments, the length of the duplex region varied from 16 bp to 20 bp. Products from unwinding reactions were separated by native PAGE and representative gel images are shown in Figure 2A. The left panel of the gel represents unwinding of the ss/ds junction and the right panel is that of a DNA fork. The experimental data for all substrates were fit to Scheme 1 using KinTek Explorer (60), and the resulting graphs are shown in Figure 2B–D with kinetic parameters listed in Table 2. Three observations are pertinent here. First, unwinding of forked DNA yielded a marked enhancement in product formation (∼20%) over that seen with the ss/ds junction substrate regardless of the length of the overhang or the duplex. Second, the lag phase was reduced in the case of the DNA fork, which required only two kinetic steps to fit the data compared to the ss/ds junction substrates which required three steps. Third, there was no significant difference in the rate constant for unwinding of these substrates (*k_u_*, Table 2). The average kinetic step size, *m*, of Dda for different lengths of dsDNA is 3.4 ± 0.9 bp, when spontaneous melting of the final 8 bp of substrate (*L*_0_ = 8) (18) and melting at the junction of the fork due to helicase binding (*L*_B_ = 2, described below) are taken into consideration.

The model in Figure 2E indicates the possible binding modes of Dda to the ss/ds junction substrate or the DNA fork substrate when considering only the 7 nt overhang. Only one ssDNA binding site exists for the ss/ds junction whereas two sites are available on the DNA fork. Dda can bind to each arm of the fork independently, according to the model. Under conditions of saturating enzyme concentration, both arms of the fork can bind to Dda.

### Dda can bind to the 5′-overhang or 3′-overhang of the DNA fork

**Figure 3.**
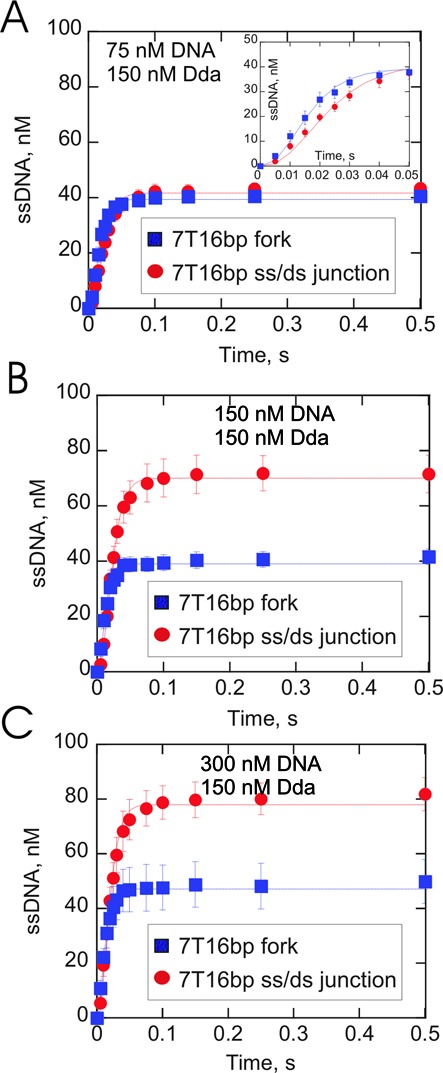
DNA unwinding under conditions in which the DNA concentration increased while the helicase concentration was held constant. **(A–C)** Dda-catalyzed unwinding of 7T16bp ss/ds junction or forked DNA substrate (Table 1). Dda (150 nM) was pre-incubated with substrate (75 nM, 150 nM or 300 nM DNA) prior to initiation of the reaction by mixing with ATP and Mg^2+^. The concentration of product was similar for each concentration of DNA fork (blue squares) at 75 nM (A), 150 nM (B) and 300 nM (C) Dda. However, the concentration of product for the ss/ds junction substrate (red circles) increased when the DNA concentration was increased.

The data in Figure 2 indicated that a DNA fork substrate was unwound somewhat better than an ss/ds junction under conditions where enzyme can saturate the ssDNA binding sites. The concentration dependence of this observation was tested by performing unwinding experiments at fixed enzyme concentration (150 nM) and increasing substrate concentrations (75–300 nM). When enzyme concentration exceeded DNA concentration, similar unwinding curves were observed for both substrates (Figure 3A). However, when the DNA concentration was equal to (Figure 3B) or greater than enzyme concentration (Figure 3C), the ss/ds junction substrates were unwound to a greater extent than the forked DNA. Hence, the quantity of product formation was reversed under conditions in which the substrate concentration exceeded the enzyme concentration (i.e. the ss/ds junction produced more product than the DNA fork). The unwinding rates for each condition were ∼250 bp/s (Table 3). A subtle difference was observed in the lag phase, which was reduced for the DNA fork compared to the ss/ds junction. The lag phase results were similar to that observed in Figure 2, and consequently, fewer steps were required for to fit the unwinding data for the DNA fork under excess substrate conditions.

In order to explain how Dda might yield more product with the ss/ds junction under conditions of excess substrate, we considered a model in which Dda might bind to the fork in two modes as shown in Figure 2E. Dda is a 5′-3′ helicase that could load on to the 3′ strand instead of the 5′ strand, resulting in non-productive binding. In addition, two Dda molecules could load onto to the same DNA fork as shown in Figure 2E, resulting in one molecule bound in a non-productive manner. Under all conditions, Dda can bind to the ss/ds junction substrate in only one manner, leading to productive binding, as shown in Figure 2E. However, Dda can bind to the DNA fork in an unproductive manner, which could result in reduced product formation when the substrate concentration is in excess. Under conditions in which the Dda concentration was in excess to the DNA, all ssDNA binding sites would be bound, therefore all substrates would have Dda bound to the appropriate overhang.

Binding of Dda to the DNA fork and the ss/ds junction is summarized by the scheme in Figure 2E. In order to determine if this model could account for the amplitudes observed under the different conditions in Figures 2 and 3, KinTek Explorer (60) was used to simulate the concentration of all the possible species that could be formed when Dda binds to varying concentrations of DNA (Supplementary Figure S3). The amount of ssDNA product formed from the productively-bound species was also determined. The model takes into account the processivity for each species, which can be estimated from the data in Figure 2 (Table 2). The predicted and the observed concentrations of the product formation were found to be similar (Supplementary Figure S3). Therefore, the distribution of Dda on the DNA fork due to binding to the 3′-overhang in a non-productive manner explains the quantity of product observed for the DNA fork and the ss/ds junction at different DNA concentrations. Hence, the relative amounts of product of the DNA fork and the ss/ds junction can be explained to a large degree by accounting for productive and non-productively bound enzyme at different enzyme and substrate concentrations. We next turned our attention to understanding why Dda unwinds the DNA fork with greater processivity and a reduced lag phase.

### DNA footprinting with potassium permanganate results in a different pattern of protection for the DNA fork compared to the ss/ds junction substrate

Interaction between Dda and DNA was investigated by DNA footprinting using KMnO_4_, which reacts much more readily with thymidine bases in ssDNA compared to duplex DNA or protein bound to ssDNA (63,65,66). The interaction of Dda with each DNA strand (tracking and displaced) was studied using forked and ss/ds junction substrates. The substrates used were similar and the conditions of DNA footprinting were otherwise identical to the unwinding assays. The ss/ds junction substrate contained a ssDNA region with seven thymidine residues flanked on either side by duplex with one or more thymidine residues in the duplex region (Table 4). The forked substrate had an additional seven thymidine residues on the displaced strand.

**Figure 4.**
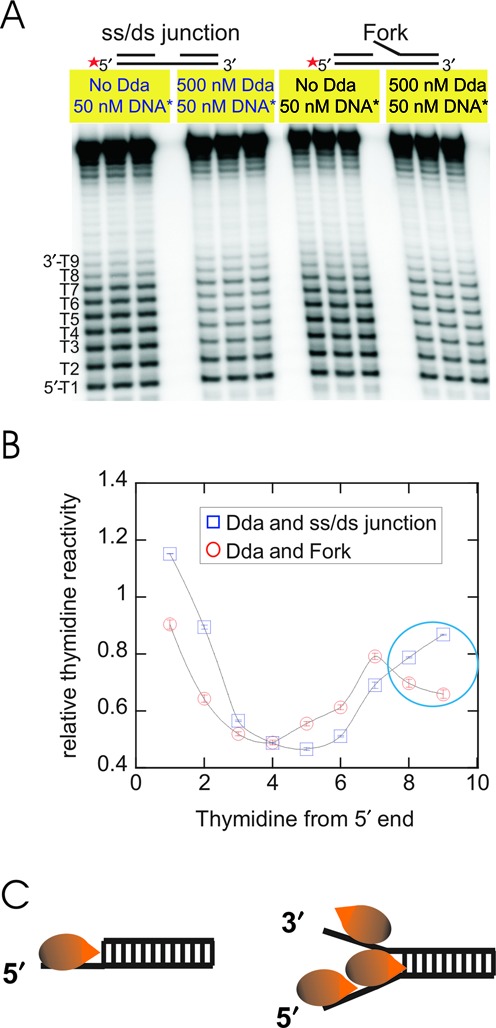
Potassium permanganate (KMnO_4_) footprinting of DNA in the presence of Dda helicase. **(A)** DNA sequences are shown in Table 4. A footprinting gel of an ss/ds junction substrate compared to a forked substrate in the absence and in the presence of Dda. Radiolabeled DNA (50 nM) and Dda (500 nM) were incubated at 25°C for 1 min followed by reaction with KMnO_4_ (5 mM) for 5 s. The reaction was quenched using 1M βME and 0.2 M EDTA. After treatment of DNA with piperidine to cleave the DNA, fragments were resolved on a 20% acrylamide, 7M urea gel. Radioactivity was visualized using a PhosphorImager, and the relative thymidine reactivity was determined. **(B)** Quantitative analysis of the footprinting of ss/ds junction and forked DNA substrates. The intensity of the thymidine reactivity in each band is expressed as a fraction of the total amount of radioactivity present in the reaction. The relative thymidine reactivity is obtained by dividing the reactivity in the presence of Dda by the reactivity in the absence of Dda. The numbered thymidines in panel A correspond to the numbers in Table 4 and are plotted in the graph. Data are from three separate experiments and the standard deviations are shown (error bars are within the points). **(C)** Cartoon depicting the species present at saturating Dda concentrations.

Permanganate footprinting was performed under excess enzyme conditions as described (Materials and Methods). Figure 4A shows the image of the denaturing PAGE, indicating the reactive thymidine residues in the tracking strand. Experiments were performed in triplicate, and the relative reactivity for each position was determined in the absence or presence of Dda in order to compare the change in thymidine reactivity. Quantitative analysis of the gel revealed a distinct pattern of reactive and protected regions in the presence of Dda. The resulting footprint was consistent with binding of Dda to the ssDNA (Figure 4B). Reactivity at thymidine position 1 was highest, indicating that this position was least protected upon binding by Dda. The reactivity was reduced in the middle portion of the binding site (thymidine positions 3 through 6). The footprinting pattern is consistent with binding of Dda, resulting in protection of thymidines, centered in the ssDNA region.

The reactivity of thymidines at positions 8 and 9 indicate the primary difference between the ss/ds junction and forked DNA substrates. These positions exhibit lower reactivity for the DNA fork compared to the ss/ds junction (circled region in Figure 4B). The lower thymidine reactivity is likely due to these residues being sequestered by Dda, and therefore being protected from reaction with KMnO_4_. The results indicate that Dda binds differently to DNA fork compared to the ss/ds junction, with the only difference in the substrate being the presence or absence of the displaced strand.

The displaced strand was examined directly for binding by permanganate footprinting. The conditions were the same as those used for footprinting of the tracking strand (500 nM Dda, 50 nM DNA). The resulting thymidine reactivity of the displaced strand indicated a similar pattern of protection as observed in the tracking strand of the ss/ds junction (Supplementary Figure S1). This protection pattern suggests that a molecule of Dda is bound to the displaced strand under saturating enzyme conditions. Hence, under conditions of excess enzyme concentration relative to substrate binding sites, Dda can protect both strands of the forked substrate. The footprinting pattern on the fork suggests that three Dda molecules may be bound to the fork, one protecting thymidines 2–6, another protecting thymidines 8–9, and possibly extending into the duplex region of the substrate, and one on the displaced strand (Figure 4C). This can explain the higher amplitude and the faster lag phase for unwinding of the fork when enzyme concentration is in excess of substrate concentration.

### Neutralization of the negative charges on the DNA phosphate backbone alters its interaction with Dda helicase

**Figure 5.**
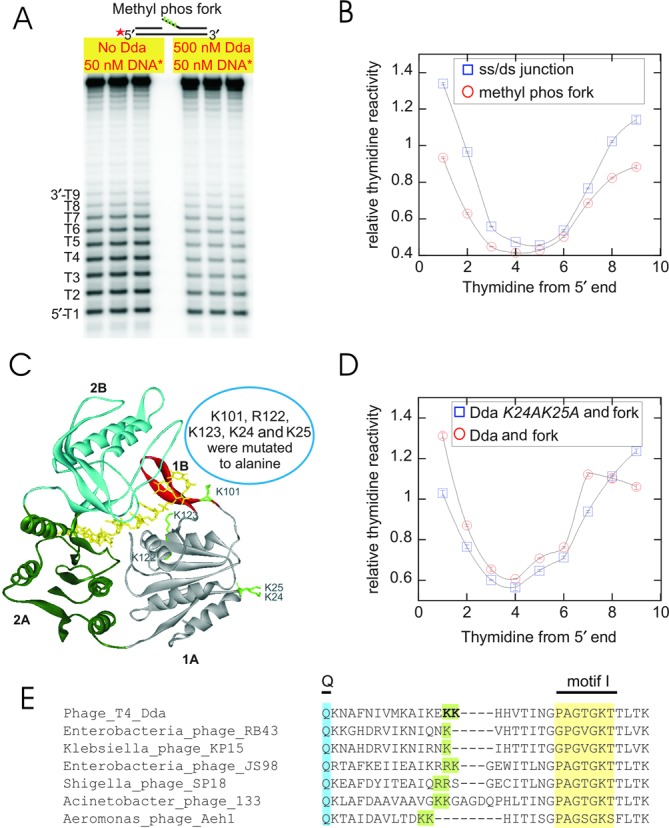
DNA footprinting with methyl-phosphonate substrate and mutated Dda. **(A)** Gel image of the methyl-phosphonate modified DNA substrate after permanganate footprinting in the presence or absence of Dda. **(B)** Quantitative analysis of the DNA footprinting gel indicating the thymidine reactivity (ss/ds junction (blue), MeP fork (red)). **(C)** Structure of Dda helicase bound with ssDNA (PDB code 3UPU (28)). Domains are colored as follows: 1A colored gray, 1B colored red, 2A colored green and 2B colored blue. The figure shows the lysine residues that are mutated to alanines (K24, K25, K101, R122 and K123). The bound DNA is colored yellow. **(D)** Footprint of the forked DNA substrate in the presence of wtDda (red) and Dda *K24A-K25A* (blue). The footprint pattern of the mutant is similar to that of the MeP-modified fork with wtDda, exhibiting an increased reactivity of the eighth and ninth thymidines (B). The data represent the average of three separate experiments with standard deviations. **(E)** There is a conservation of positive charges in the vicinity of K24 and K25 (green) in Dda proteins from T4-like phages. The Q motif (blue) and motif I (yellow) are also shown.

The possibility that the interactions between the displaced strand and Dda occur through electrostatic interactions was considered. To explore this possibility, the electrostatic interactions with the phosphate backbone were removed by creating modified oligonucleotide substrates (18,67–69) using methyl phosphonate. Replacing phosphoryl oxygen atoms with methyl groups confers loss of negative charges to the DNA substrate. The MeP-modified fork had four MeP residues on the displaced strand (Table 4). Permanganate footprinting of the modified substrate was performed and the results indicated that insertion of MeP moieties in the fork altered its binding interaction with Dda (Figure 5A). The increased permanganate reactivity at thymidine positions 8 and 9 in the MeP-modified fork follows the same pattern as observed with the ss/ds junction substrate (Figure 5B). A straightforward interpretation of these results is that Dda is unable to interact with the displaced strand of the fork due to the MeP modifications. The resulting footprint pattern of the MeP DNA fork is similar to that of the ss/ds junction substrate, compared to the unmodified fork (Figure 4B), which emphasizes the importance of electrostatic interactions between Dda and the displaced strand.

### Mutation of lysine residues in Dda helicase reduces its ability to interact with the displaced strand of the DNA fork substrate

We sought to explore the sites of interaction between Dda and the substrate. The crystal structure of Dda (28) exhibited several lysine and arginine residues in domains 1A and 1B that were proposed to form a binding site for duplex DNA (Figure 5C). Initially, three amino acids at the base of the beta-hairpin were mutated to form the triple mutant, *K101A-R122A-K123A*. This mutant enzyme exhibited ssDNA-stimulated ATPase activity, but no DNA unwinding activity was observed under single-turnover conditions (Supplementary Figure S2). We reasoned that this area of the protein was critical for separation of duplex DNA due to its close proximity to the pin domain (28). Therefore, a double mutant was created in which positively charged residues were selected further away from the pin, but within the region proposed to interact with the duplex (Dda *K24A-K25A*, Figure 5C).

The double mutant exhibited robust DNA unwinding activity (see below). Dda *K24A-K25A* was examined by DNA footprinting with the DNA fork substrate (Figure 5D). Dda *K24A-K25A* binding resulted in a footprinting pattern in which thymidine positions 8 and 9 were more reactive than observed with wtDda. Hence, the KMnO_4_ protection pattern of *K24A-K25A* was similar to that observed with the ss/ds junction substrate in the presence of wtDda. We conclude that the reactivity pattern of wtDda with the DNA fork results in specific interactions that lead to reduction of reactivity at thymidine positions 8 and 9. This pattern requires interaction with the displaced strand because it is not observed with the ss/ds junction or with the MeP substrates. The reduced reactivity at positions 8 and 9 may also require interaction with the duplex DNA, as illustrated by the results with the *K24A-K25A* double mutant (Figure 5D). We cannot eliminate the possibility that this region of the protein is interacting with the displaced strand. However, we favor a model in which K24 and K25 interact with the duplex based on the path of the duplex DNA in the structures of UvrD (70) and PcrA (35). Overall, the results are consistent with a model in which optimal unwinding occurs when Dda interacts with a DNA fork through three ‘points of contact’: (i) the tracking strand, (ii) the duplex near the junction and (iii) the displaced strand. The importance of these residues is further illustrated by the conservation of positively charged residues in this region among Dda helicases from T4-like phages (Figure 5E).

### Dda *K24A-K25A* exhibits a different lag phase for unwinding of a DNA fork compared to a ss/ds DNA junction

**Figure 6.**
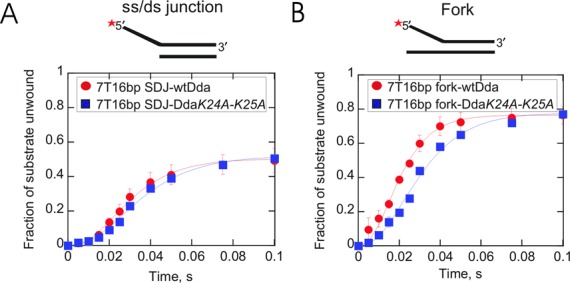
Unwinding of 7T16bp ss/ds junction or forked DNA substrates in the presence of Dda or Dda *K24A-K25A*. Dda was pre-incubated with DNA in the reaction buffer and rapidly mixed with equal volume of 5 mM ATP, 10 mM Mg(OAc)_2_, protein trap (5 mM) and annealing trap (150 nM) in the same buffer for the times indicated. **(A)** DNA unwinding of the ss/ds junction substrate in the presence of Dda or Dda *K24A-K25A*. Panel **(B)** shows unwinding of the Fork substrate by Dda or Dda *K24A-K25A*. There is no significant difference in the rate or amplitude for unwinding of the ss/ds junction substrate by wt Dda compared to the mutant Dda (panel A). In panel B, the amplitude for DNA unwinding of the forked substrate is the same for wt and mutant Dda. Unwinding of the fork by mutant Dda exhibits a longer lag phase when compared to the unwinding of the fork with wtDda.

Since the Dda double mutant, *K24A-K25A* exhibited differences in binding to the fork DNA, we examined its ability to unwind DNA substrates under single-turnover conditions. The double mutant exhibited a very similar kinetic progress curve during unwinding of the ss/ds junction substrate when compared to wtDda (Figure 6A). However, the progress curve for unwinding of the DNA fork exhibited a significantly longer lag phase for Dda *K24A-K25A* compared to wtDda (Figure 6B). The amplitude for Dda *K24A-K25A* was identical to that of wtDda. Therefore, disruption of the putative interaction with the duplex through mutation of the two lysine residues leads to DNA unwinding curves that differ from wtDda for the DNA fork but not the ss/ds junction.

The results from DNA unwinding and DNA footprinting for Dda *K24A-K25A* can be interpreted in terms of the number of available sites of interaction between the enzyme and the substrate. In addition to the required interaction (for unwinding) of Dda with the 5′-arm of the substrate, Dda can interact with the 3′-arm of the fork as well as the duplex region. Only when all three interactions occur can optimal DNA unwinding occur, marked by a reduced lag phase and greater amplitude for the DNA fork. When Dda initiates unwinding from a substrate in which one of these interactions is lost, then the progress curve for DNA unwinding is altered. Removal of the 3′-arm of the DNA fork or disruption of the interaction with duplex can reduce the amplitude and increase the lag phase.

### Interaction with the displaced strand during duplex DNA unwinding requires a DNA fork greater than 6 nt in length

**Figure 7.**
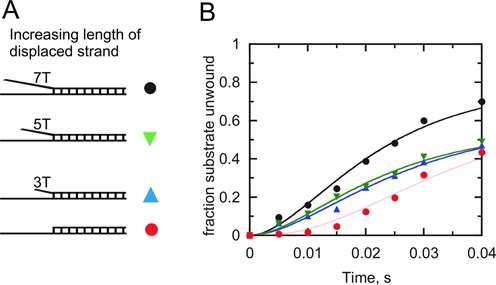
Dda-catalyzed unwinding of DNA substrates with increasing 3′-ssDNA tail length. **(A)** The substrates contained 0, 3, 5 or 7 thymidines in the 3′-ssDNA overhang. Dda (150 nM) was incubated with DNA substrates at (2 nM) and unwinding was initiated by the addition of ATP (5 mM). **(B)** The quantity of ssDNA is plotted for the early portion of the progress curve. The substrates containing 3, 5 or 7 nt in the overhang were fit to a two-step kinetic mechanism whereas the data for the substrate with no overhang was fit to a three-step mechanism.

In order to learn whether the length of the displaced strand is critical during Dda-catalyzed duplex DNA unwinding, DNA substrates with increasing 3′-ssDNA tail length were used in single-turnover unwinding experiments. The substrates were designed to contain 0, 3, 5 or 7 thymidines in the 3′ssDNA region adjacent to the 16 bp duplex (Figure 7A). The unwinding reactions were carried out under excess enzyme conditions. The resulting data illustrate the reduction in the lag phase as the 3′-arm of the fork increases in length (Figure 7B). The amplitude also increases for the forked DNA (seven thymidines in the 3′-ssDNA region) substrate. The reduced lag phase for the DNA fork compared to the ss/ds junction suggests that fewer steps are needed to unwind the fork, even though each substrate contains the same number of base pairs. We conclude that Dda interacts with the DNA fork in a manner that disrupts the first 2–3 base pairs, thereby reducing the number of base pairs needed for DNA unwinding. This is consistent with footprinting results (Figure 4B) which suggest that two Dda molecules may be bound to the tracking strand of the forked substrate, with the leading Dda molecule invading the duplex region as depicted in Figure 4C.

**Figure 8.**
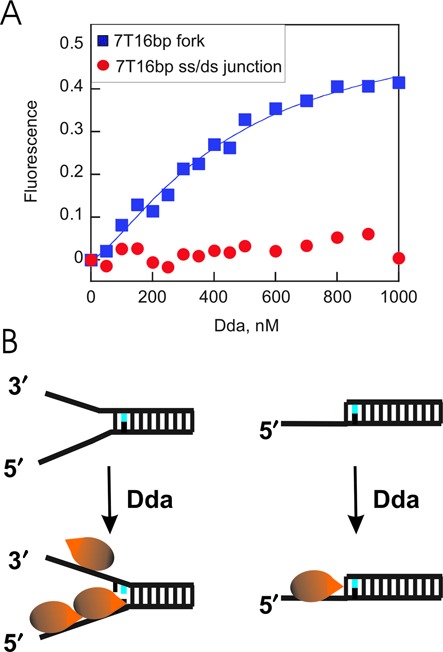
Titration of 2-aminopurine labeled DNA with Dda. **(A)** 2-aminopurne labeled fork and ss/ds junction DNA were titrated with Dda and the fluorescence was monitored at 380 nm. Data was fit to the Hill equation to obtain a Hill coefficient of 1.9 ± 0.2. The Dda concentration at which half of the maximal 2-aminopurine fluorescence was reached was 330 ± 40 μM. Duplicate experiments produced similar results. Values are the average of two experiments; errors are the standard error of the fit. **(B)** Model illustrating melting of 2–3 bp of the 2-aminopurine (blue) labeled fork upon binding by Dda but no melting upon binding of the ss/ds junction DNA.

We tested the model for melting of the DNA fork by placing a fluorescent nucleotide, 2-aminopurine (2-AP), into the DNA substrate. Titration of the 2-AP fork with Dda leads to an increase in fluorescence, consistent with loss of base pairing of the 2-AP (Figure 8A). In contrast, no increase in fluorescence was observed with the 2-AP ss/ds junction, indicating that 2-AP remains base paired. The Hill coefficient of 1.9 ± 0.2, along with the high concentration of Dda needed to approach saturation, is consistent with multiple binding events as depicted in Figure 8B and supports conclusions drawn from unwinding and footprinting data.

## DISCUSSION

Helicase-catalyzed DNA unwinding can be understood in terms of the kinetic progress curve for melting of short duplexes. The progress curves contain information about the number of steps needed to unwind the substrate, the rate of each step (melting) and the dissociation rate from the substrate. The lag phase of an unwinding progress curve provides indirect information on the number of kinetic steps required to completely melt a duplex (61,71). The lag phase is observed when multiple unwinding steps are required to observe product formation and can be measured as a function of the length of the DNA duplex (62,71–76). The amplitude of the DNA unwinding curve reveals the balance between the rate for unwinding and the rate of dissociation from the DNA. For a relatively non-processive helicase, such as Dda, if the duplex length is increased from 16 to 20 bp, the fraction of substrate unwound (amplitude) is reduced (Figure 2B and D).

Comparison of the rate and processivity between two substrates can reveal which substrate is best utilized by a helicase. However, the conditions of the experiment can dramatically affect the outcome. The amplitude for Dda-catalyzed unwinding of a DNA fork is greater compared to the ss/ds junction when enzyme concentration exceeds substrate concentration. In contrast, the ss/ds junction provides a higher amplitude under conditions in which substrate concentration exceeds enzyme concentration (Figure 3C). The unwinding results under different enzyme concentrations relative to substrate concentrations can be explained based on the equilibrium binding model shown in Figure 2E and the simulation in Supplementary Figure S3. The DNA fork has two binding sites, the 5′-arm and the 3′-arm. Dda can bind to either site, but binding only to the 3′-arm results in a non-productive complex because Dda is a 5′-3′ helicase. Binding to the non-productive site of the DNA fork leads to less product when substrate concentration exceeds enzyme concentration (26). The ss/ds junction with the 5′-overhang has only one binding site which results in a productive complex. These results indicate the importance of examining a substrate under conditions of varying enzyme concentration relative to the substrate concentration in order to determine which substrate is used more efficiently.

An interesting observation for the DNA fork is that the lag phase was reduced when compared to the ss/ds DNA junction (Figure 2). The importance of the interaction with the displaced strand is further revealed by examining the lag phase of substrates in which the length of the displaced strand overhang is increased (Figure 7). The reduction in the lag phase can be explained if interaction between Dda and the displaced strand results in re-positioning of Dda at the fork resulting in melting of a few bps (Figure 4C). The kinetic step size of the helicase determines the number of reaction cycles necessary for product formation. Fitting the unwinding data required a three-step mechanism for the ss/ds junction; whereas a two-step mechanism was sufficient to fit the data for the DNA fork (Figures 2, 3 and 7). The reduced number of kinetic steps implies that binding of Dda to the fork results in melting of a sufficient number of base pairs as to reduce the number of steps needed to unwinding the fork. Thus, the higher amplitude of product formation and the reduced lag phase for unwinding of forked DNA could be due to interaction of Dda with each strand of the fork which results in melting 2–3 base pairs. For the forked substrate, when calculating the unwinding rate (Tables 2 and 3), the number of base pairs at the junction which melt due to binding of Dda must be considered.

**Figure 9.**
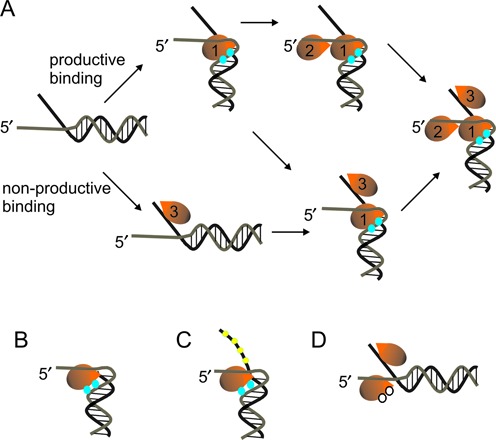
Model for interaction between Dda and DNA fork substrates when enzyme is in excess. **(A)** A Dda molecule (1) binds to the tracking strand (gray) in the known binding site (28) while the displaced strand (black) interacts with a second site on the surface of the enzyme. The duplex interacts with residues on domain 1A, with two lysine residues (K24 and K25) illustrated by the blue circles. The translocase strand is proposed to make a ∼90° turn upon entering the ssDNA binding site. The three sites of interaction are proposed to be necessary for the observed destabilization of the duplex due to helicase binding. This melting of 2–3 base pairs provides sufficient space for a second Dda molecule (2) to also bind to the tracking strand. No preference for binding to the 3′-overhang or 5′-overhang has been observed, so binding of the Dda molecule labeled 3 (on the displaced strand) can occur before or after the Dda molecules labeled 1 and 2. In the absence of the displaced strand **(B)** or in the presence of the methyl-phosphonate modified displaced strand **(C)** duplex destabilization does not occur and only a single Dda molecule can bind to the substrate. **(D)** The interaction with the duplex region is lost upon mutation of the lysine resides at positions K24 and K25, illustrated by the white circles.

DNA footprinting was performed to provide evidence for destabilization of the duplex with a fork substrate. The DNA fork substrate clearly shows an altered footprinting pattern compared to the ss/ds DNA junction (Figure 4). The reduced reactivity of thymidines at positions 8 and 9 could be due to Dda binding which could protect the thymidines from KMnO_4_. We propose a model whereby Dda interacts with the fork through three regions of contact: the tracking strand, the duplex and the displaced strand (Figure 9). Interaction with all three regions results in destabilization of 2–3 base pairs. This accounts for the observed differences in the kinetics as well as the DNA footprinting and 2-AP fluorescence for the DNA fork compared to the ss/ds junction. If the displaced strand ssDNA overhang is removed, then the incoming DNA interacts only with the translocase strand and the duplex (Figure 9B), thereby removing the destabilization of 2–3 base pairs. The interaction with the displaced strand is proposed to occur primarily through electrostatic interactions with the phosphate backbone, based on the loss of the DNA-footprinting pattern in the presence of the methyl-phosphonate DNA (Figure 9C). Loss of the interaction with the duplex also relieves the destabilization at the fork, as shown by mutagenesis of key lysine residues in domain 1A (Figure 9D). Hence, three regions of contact between Dda and a DNA fork result in destabilization of the fork, leading to the reduced lag phase and increased amplitude for DNA unwinding.

These results are consistent with Dda being an ‘active’ helicase, which engages both strands of the substrate and actively melts the base pairs rather than relying on thermal fraying to unwind the duplex (27,28). The destabilization of duplex DNA due to binding of Dda helicase is reminiscent of the interaction of primer/template junctions with DNA polymerases (77,78). The template DNA strand is often bent by ∼90° at the active site of polymerization, which exposes the template base to the incoming nucleotide. In the case of DNA helicases, X-ray crystallographic structures of PcrA helicase indicate a ∼90° bend in the tracking strand at the site of DNA melting (35). We suggest that the interaction with the three DNA regions, duplex, tracking strand and displaced strand causes torsion in the duplex resulting in melting of the base pairs. Binding of the displaced strand by Dda also leads to the observed ‘pausing’ in the kinetic mechanism described in previous reports (18,27). The specific path of the displaced strand on the surface of Dda is yet to be determined. It is possible that Dda guides the displaced strand into gp32, which is known to bind to Dda. The specific effect of gp32 on Dda-catalyzed unwinding remains to be explored.

The best characterized ‘helicase machine’ in terms of the pathway for each arm of the DNA fork is the RecBCD helicase. The structure of the RecBCD complex provided the first molecular view of an SF1B helicase (79). The RecD2 structure shows interactions with the tracking strand, but interactions with the displaced strand have not been visualized (51). The X-ray structure of RecBCD illustrates melting of several base pairs due to binding interactions at the DNA fork. DNA footprinting with KMnO_4_ also indicated that RecBCD helicase melts a few bases of duplex upon binding (80).

Biochemical (81) and electron microscopy (82) evidence for the superfamily four helicases suggested that a single strand of DNA passes through the channel with the second strand passing outside of the ring. In the ‘direct exclusion’ model, regions of the helicase act as a mechanical wedge, stripping the strands apart as one strand translocates through the center (83). Evidence has been provided for the specific pathway taken by the displaced strand through a specific channel on the outside the MCM hexameric helicase (84). Mutations in a positively charged patch on the exterior surface destabilized the interaction with the 5′-arm of a DNA fork and reduced DNA unwinding.

The variants described here with mutations in domain 1A (Figures 5 and 6) provide the first evidence suggesting that Dda binds to the duplex region in addition to the ssDNA tracking strand. Removal of the proposed interactions by mutating positively charged residues to neutral residues eliminates (Supplementary Figure S2) or alters DNA unwinding activity (Figure 6). This supports the idea that the interaction contributes to the unwinding activity of Dda. In the case of SF1A helicases, which translocate from 3′ to 5′, interaction of the duplex with domain 1B has been shown by X-ray crystallographic studies (35,70). However, deletion of domain 2B actually enhances helicase activity for the *Escherichia coli* Rep helicase (85,86). Therefore, specific interactions with duplex DNA can have varying outcomes for different helicases in terms of the impact on DNA unwinding.

Dda is known to interact with the single-stranded binding protein, gp32 (87). It is possible that the displaced strand feeds directly into gp32 after being melted. Hence, the interaction between Dda and the displaced strand is likely to be transient. As Dda moves along the tracking strand, the displaced strand must also be ‘translocated’ across the surface of the helicase. In the case of UvrD helicase, translocation of the displaced strand has been examined directly. UvrD monomers bound to a 5′-ss/ds junction, with the overhang on the displaced strand due to UvrD's 3′–5′ directionality, have been shown to translocate away from the dsDNA (26). Single molecule approaches revealed UvrD helicase switching strands during unwinding and translocating backward on the displaced strand (25).

Although monomeric Dda has been shown as an efficient helicase (47), there remains the possibility for dimerization under conditions that bring two molecules together. Although the crystal structure of Dda revealed a monomeric enzyme when bound to ssDNA (28) and a monomer is capable of unwinding DNA (47), multiple Dda molecules are needed for optimal unwinding of a fork substrate. Indeed, footprinting experiments reveal binding of a molecule of Dda to the 3′-arm and possibly two molecules to the 5′-arm of the DNA fork (Figure 4C). It is known that Dda interacts with gp32 which can significantly enhance DNA replication during strand displacement synthesis *in vitro*. A model put forth by the Morrical lab, the mixed oligomer model, illustrated that Dda-gp32 interactions induce the oligomerization of Dda, forming a mixed oligomer of both proteins (87). Work here sets the stage for examining the handoff of ssDNA from Dda to gp32 at a DNA fork.

## SUPPLEMENTARY DATA

Supplementary Data are available at NAR Online.

SUPPLEMENTARY DATA
