# Supplementary material for: Simultaneous binding to the tracking strand, displaced strand and the duplex of a DNA fork enhances unwinding by Dda helicase
Source: Nucleic Acids Res. 2014 Sep 23;42(18):11707–20. doi: 10.1093/nar/gku845 (PMC4191417; doi:10.1093/nar/gku845)
Supplement: SUPPLEMENTARY DATA [file supp_42_18_11707__index.html]

Simultaneous binding to the tracking strand, displaced strand and the duplex of a DNA fork enhances unwinding by Dda helicase — Simultaneous binding to the tracking strand, displaced strand and the duplex of a DNA fork enhances unwinding by Dda helicase — SUPPLEMENTARY DATA 

# Simultaneous binding to the tracking strand, displaced strand and the duplex of a DNA fork enhances unwinding by Dda helicase

## SUPPLEMENTARY DATA

**Files in this Data Supplement:**

- SUPPLEMENTARY DATA
