# Supplementary material for: Simultaneous binding to the tracking strand, displaced strand and the duplex of a DNA fork enhances unwinding by Dda helicase
Source: Nucleic Acids Res. 2014 Sep 23;42(18):11707–20. doi: 10.1093/nar/gku845 (PMC4191417; doi:10.1093/nar/gku845)
Supplement: SUPPLEMENTARY DATA [file supp_gku845_nar-00355-m-2014-File011.pdf]

Simultaneous binding to the tracking strand, displaced strand, and the duplex of a DNA fork enhances unwinding by Dda helicase

Suja Aarattuthodiyil, Alicia K. Byrd and Kevin D. Raney*

Department of Biochemistry and Molecular Biology, University of Arkansas for Medical Sciences, Little Rock, AR, 72205, USA

* To whom correspondence should be addressed. Tel.: 501-686-5244; Fax: 501-686-8169; Email: raneykevind@uams.edu

SUPPLEMENTARY DATA

CONTENTS

Supplementary Figure 1. Potassium permanganate ($KMnO_4$) footprinting of the radio-labeled displaced strand in a forked DNA substrate.

Supplementary Figure 2. Comparison of the ATPase and helicase activities of wtDda and a variant form of Dda.

Supplementary Figure 3. The concentration of each of the different species and the subsequent ssDNA that could be formed when 150 nM Dda binds to 75/ 150/ 300 nM ss/ds junction or forked DNA substrates.

Supplementary References

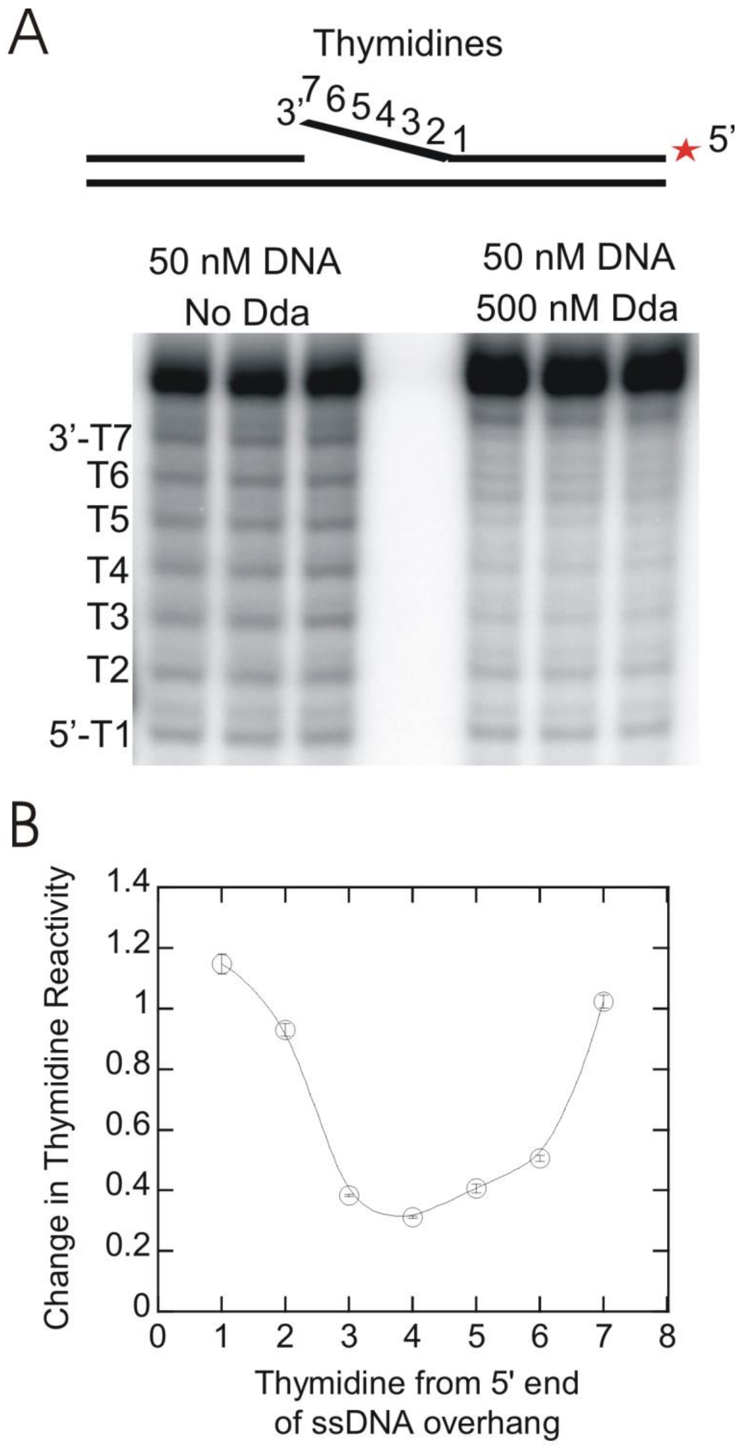

Supplementary Figure 1. (A) Potassium permanganate ($KMnO_4$) footprinting of the radio-labeled displaced strand in a forked DNA substrate. The gel images show the footprints of forked DNA substrate in the absence and presence of Dda. The thymidines in the substrate are numbered starting from the 5' end of the ssDNA overhang. **(B)** The relative reactivity of the thymidines is quantified using ImageQuant and plotted using Kaleidagraph software. The footprint pattern is in agreement with the binding of one Dda molecule to the displaced strand of the forked DNA. The data represent the average of three separate experiments with standard deviations.

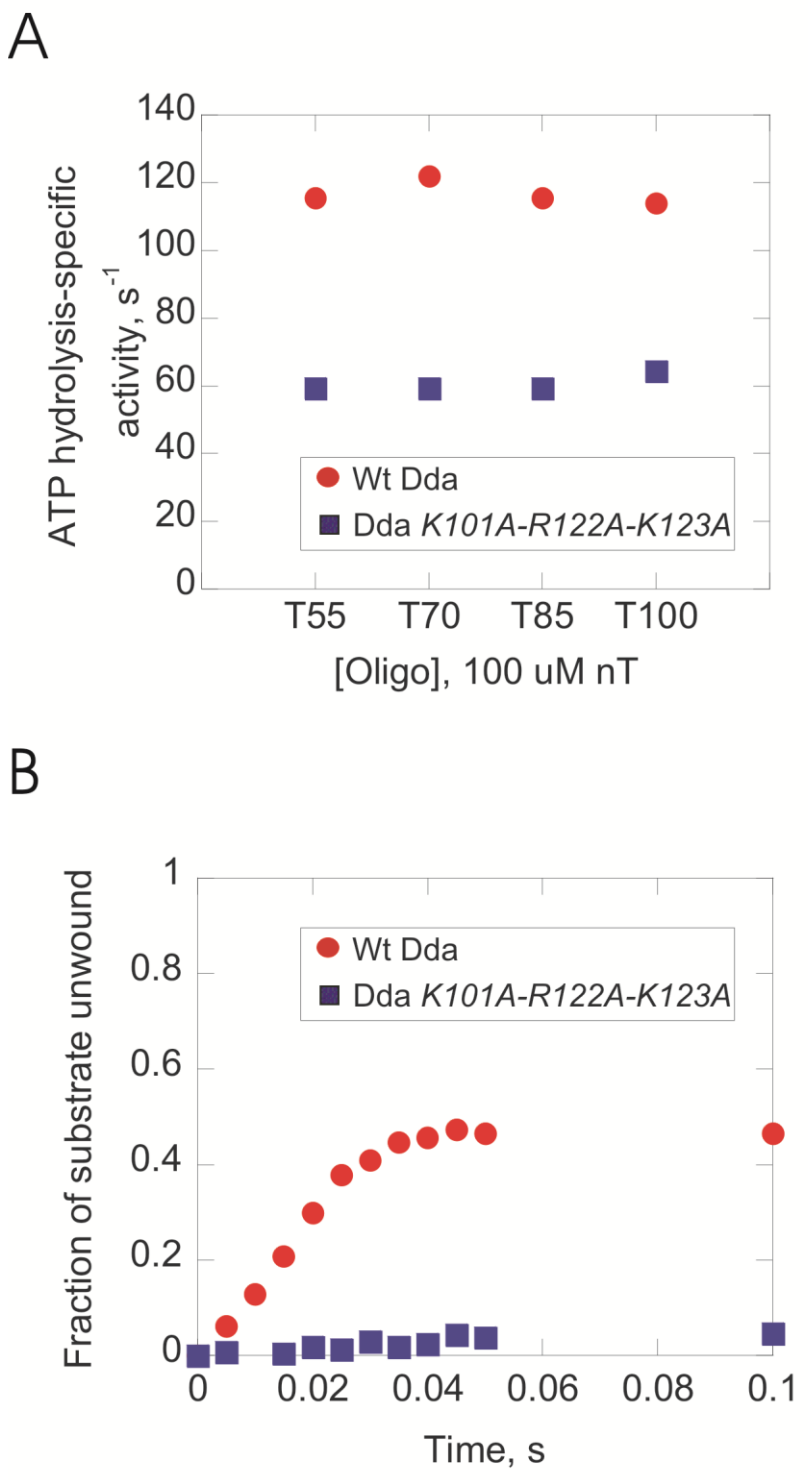

Supplementary Figure 2. Comparison of the **(A)** ATPase and **(B)** helicase activities of wtDda and a variant form of Dda where three amino acid residues near the pin region were replaced with alanines (Dda *K101A-R122A-R123A*). Although the ATPase activity of the mutant was within two fold of the wtDda, under single turn over conditions, there was no significant unwinding.

A

DNA substrates	75 nM DNA	150 nM	300 nM
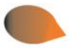	83	34	10
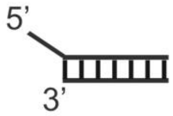	8	34	155
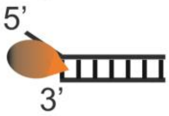	67 (40.2)	116 (69.6)	145 (87)
Total	40.2	69.6	87

B

DNA substrates	75 nM DNA	150 nM	300 nM
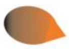	34	9	3
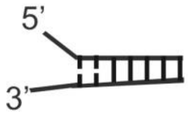	4	42	171
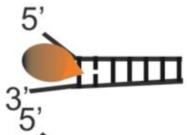	13 (7.8)	37 (22.2)	55 (33)
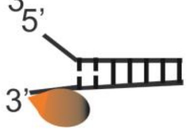	13	37	55
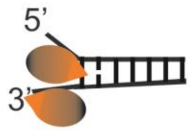	45 (36)	33 (26.4)	18 (14.4)
Total	43.8	48.6	47.4

Supplementary Figure 3. Random binding of Dda to the 3’- or 5’- ssDNA overhang(s) accurately predicts the product formation for unwinding of the ss/ds junction and fork substrates. The concentration of each of the different species and the subsequent ssDNA product that could be formed when 150 nM Dda binds to 75/ 150/ 300 nM **(A)** ss/ds junction or **(B)** forked DNA substrates as determined by kinetic simulations using the KinTek Explorer program (60). The amount of ssDNA product formed from each productively-bound species is shown in parenthesis and the total amount of product formed from all productively-bound species is shown at the end of each column. For the ss/ds junction substrate, only 60% of bound species results in ssDNA product due to the relatively low processivity of Dda (Figure 2). For the forked DNA substrate, around 60% product is formed when Dda binds only to the 5’ overhang and around 80% product is formed when Dda binds to the 5’ overhang and to the 3’ overhang (Figure 2).

SUPPLEMENTARY REFERENCES

60. Johnson,K.A., Simpson,Z.B. and Blom,T. (2009) Global Kinetic Explorer: A new computer program for dynamic simulation and fitting of kinetic data. *Anal. Biochem.*, **387,** 20-29.
